# Weight-loss strategies of South African female university students and comparison of weight management-related characteristics between dieters and non-dieters

**DOI:** 10.1186/s12889-016-3576-x

**Published:** 2016-09-01

**Authors:** Marjanne Senekal, Gabrielle L. Lasker, Lindsay van Velden, Ria Laubscher, Norman J. Temple

**Affiliations:** 1 Division of Human Nutrition, University of Cape Town, Anzio Rd, Anatomy Building Room 2.01.5, Observatory 7925, Cape Town, South Africa; 2 Biostatistics Unit, Medical Research Council, Tygerberg, South Africa; 3 Centre for Science, Athabasca University, Alberta, Canada

**Keywords:** Weight-loss strategies, Weight-loss pressure, Weight management, BMI predictors, Female students

## Abstract

**Background:**

Female university students are at risk for weight gain and use of inappropriate weight-loss strategies. By gaining a greater understanding of the weight-loss strategies used by and weight management related characteristics of these students, effective weight management interventions for this vulnerable group can be developed.

**Methods:**

Two hundred and fifty female students from South Africa universities, aged 18–25 years, participated in this cross-sectional study; 162 attempted weight loss during the year preceding the study (dieters) and 88 were non-dieters. Weight and height were measured and BMI (kg/m^2^) computed. A self-administered questionnaire was used to record all other variables. Weight loss strategies were described for dieters and compared between BMI groups within the dieters group. Weight management related characteristics were compared between dieters and non-dieters. Statistical tests included Pearson Chi-square test, independent samples *t*-test or Mann-Whitney *U* test (depending on distribution of the data). Predictors for a higher BMI and being overweight/obese (BMI ≥25 kg/m^2^) were identified using regression models.

**Results:**

Healthy weight-loss strategies included increased exercise and fruit/vegetable intake and decreased intake of sugar and fat containing items; unhealthy methods included eating little food and skipping meals; and extreme weight loss strategies included laxatives and vomiting. The most commonly used weight-loss product was Herbex. Dieters were characterized by a higher BMI, overestimation of their weight (especially normal weight students), dissatisfaction with weight and select body parts, higher intake of breakfast and healthy foods, lower intake of unhealthy foods, higher levels of vigorous physical activity, higher use of select informal weight-loss information sources and experiencing more pressure to lose weight from mothers, siblings and friends. Predictors of higher BMI and/or increased risk for BMI ≥25 included weight-loss attempt during the past year, race, dissatisfaction with waist, perception of currently being “chubby,” and higher frequencies of intake of a snack and fatty foods.

**Conclusion:**

Attempting weight-loss is common among female students and predicts BMI. Healthy (mainly), unhealthy and extreme weight loss methods are used. Dieters are characterized by a less realistic body image, lower body satisfaction, higher pressure to lose weight, use of informal weight-management information and a healthier life-style.

## Background

The prevalence of obesity (BMI ≥ 30 kg/m^2^) worldwide is continuously rising and is a global public health concern [1, 2]. Obesity peaks in middle and older age but is becoming more common at a younger age [2]. A high prevalence of overweight (BMI ≥ 25 kg/m2) (25.3 %) and obesity (21.7 %) is also seen in South African females aged 18 to 24 years [3]. It has been well documented that female students, especially those in their first year at tertiary education institutions, are prone to gaining weight [4]. Excessive body weight i.e. being overweight or obese or having central obesity is associated with serious health consequences [5, 6].

The rise in overweight and obesity has contributed to increasing numbers of people within populations who need to, and attempt to lose weight [7]. This may also be the case with university students [8, 9]. Unfortunately, the reality for most dieters is that weight-loss attempts have a poor success rate and the minority who do succeed in losing weight regain most of the lost weight within a few years [10].

It further needs to be considered that in Western society young females, including university students, often aim for the thin beauty ideal, leading them to try and attain weight goals that are unrealistically low [11–14]. To achieve this desired body weight they may be using dieting strategies that are unhealthy (e.g., fasting) or extreme (e.g., laxatives) [12, 15, 16]. Extreme concern with weight, dieting and body perception can result in high-risk behaviours such as restrictive and disordered eating, binging, purging and ultimately eating disorders [8, 15]. The high prevalence of these disorders in adolescent and young adult females is a particular concern [17, 18].

Within the South African context a further concern is that obese women, specifically black women, may be unaware of the need for weight loss for health purposes [19]. Research carried out in South Africa and other countries shows that black females are generally more tolerant of a larger body size and that this may be socially more desirable, [19–21]. A number of studies have also shown that overweight black women are more likely than white women to perceive themselves as being normal weight [12, 22, 23]. These findings provide important insights in the lack of awareness of black women in their personal weight management needs. Research on black South African female students and adult women does, however, show that acculturation to Western body shape and size ideals may be taking place [12, 24] and that dieting is not uncommon among young black women [12].

The aims of this cross-sectional survey were to investigate the weight-loss strategies used by female university students at select South African Universities who attempted weight loss over the year preceding the study (aim 1) and to compare weight related characteristics between dieters and non-dieters (aim 2). These insights will contribute to development of effective intervention strategies and public health messages to promote healthy weight management in this vulnerable population.

## Methods

### Participants

The target population for this research were female students, aged 18–25 years, attending one of three universities in the Western Cape. A convenience sampling technique was used to select the sample. Exclusion criteria were as follows: students who were pregnant or breastfeeding at the time of the study, elite athletes, were following medically-related dietary restrictions, had diseases that may affect weight and individuals with physical disabilities that may influence body shape perception and weight. A dieter was defined as a student who reported one or more weight-loss attempts during the year preceding the study, while non-dieters did not attempt weight loss during the year preceding the study. The least frequently used weight loss strategy reported by Malinauskas et al. [25], i.e. laxatives used by 3 % of their sample, was used to calculate the sample size for this study. The Sample Size Proportions option of the OpenEpi calculator (www.openepi.com) was used for these purposes with an anticipated percentage frequency of 3 % and confidence level of 2.1, which resulted in a sample size of 254. We achieved a final sample of 250. One volunteer did not meet the inclusion criteria, as she reported to have scoliosis.

### Measures

#### Weight and height

Height was measured to the nearest 0.1cm with students wearing no shoes. Students stood against the stadiometer (Leicester Height Measure MK11) facing forward with the head in the Frankfort plane. The fieldworker lowered the headpiece until it was touching the cranium and took the reading. Weight was measured on a calibrated electronic scale (SECA Robusta 183) to the nearest 0.1kg with students wearing minimal clothing and no shoes. The fieldworkers ensured that students stood facing forward on the scale with feet slightly apart, distributing weight evenly before the reading was taken. These measures were taken in a secluded place on campus or in residences to protect the privacy of participants.

BMI was computed as weight in kilogram (kg) divided by height in meter (m) squared. The following BMI categories were used for interpretation of weight status: underweight <18.5 kg/m^2^, normal weight 18.5–24.9kg/m^2^, overweight 25–29.9 kg/m^2^ and obese ≥30kg/m^2^ [26].

#### Questionnaire on weight loss strategies and weight-related characteristics

The questionnaire for this research was developed based on reported weight loss strategies and weight management-related characteristics of females extracted from the literature [9, 12–15, 18, 19, 24, 27]. Discussions with a panel of nutrition experts were conducted to finalize the conceptual framework that underpinned the study and thus the questionnaire. The draft questionnaire was checked by the expert panel for construct and content validity and completed by four female students to test readability and comprehension of the questions to ensure face validity before it was finalized.

Questions on weight loss strategies were categorized as healthy, unhealthy and extreme, with strategies included in each of these three categories adapted from Neumark-Sztainer et al. [28]). Healthy weight-loss strategies covered in the questionnaire are increased exercise, increased intake of fruit and vegetables, eating less high-fat foods and eating or drinking sugar-free versions of foods; unhealthy strategies are diet shakes, eating little food, skipping meals, fasting and smoking more cigarettes; and extreme strategies are self-induced vomiting and use of laxatives, methamphetamine (“tik”), diet pills or herbal mixtures.

Commercial weight-loss products, which had some overlap with items in the unhealthy and extreme weight loss strategy categories, were also included in the questionnaire. These included Herbex (claimed to promote fat burning), Herbalife products (includes fat burners and diet shakes), GI (Glycemic Index) Lean products (includes fat burners and diet shakes), Biomix Slimming Solution (claimed to be a metabolic accelerator and appetite suppressant), Hoodia (appetite suppressant derived from a South African cactus plant), USN (Ultimate Sports Nutrition) weight-loss products (includes fat burners, appetite suppressants and meal replacements), CLA (conjugated linoleic acid) (fatty acid that is claimed to assist with weight loss), Leanor (slimming concentrate), Simply slim (slimming mix), Phentermine (used for appetite suppression and energy burning), Sibutramine (appetite suppressant), Orlistat (reduces fat absorption) and products containing Amfepramone (strong appetite suppressant), Phendimetrazine (appetite suppressant) or Cathine (appetite suppressant). The listed products were commonly available over the counter or prescribed by medical practitioners at the time of the study. The most likely time of year to use a commercial weight loss product (winter, summer, spring or autumn) was also asked.

Weight management-related characteristics included in this paper are socio-demographic information, past pregnancy (yes or no), smoking (yes or no), current body shape perception and satisfaction, meal pattern, food choices, physical activity and environmental influences (sources of weight management information and weight loss pressure experienced).

Socio-demographic questions included age, ethnicity (black, white, mixed ancestry, Indian or other), accommodation (private residence, university residence, at home or other) and mother’s level of education (secondary school or less or tertiary education). Questions on perception of current weight (too thin, just about the right weight, too chubby) and satisfaction with weight, height, waist, hips, legs, arms, stomach, buttocks and face (satisfied, unsure or not satisfied) covered body image-related concepts. Assessment of environmental influences included questions on attaining information on weight management from magazines, television, internet, friends, pharmacist, nutrition expert, family, doctor (almost always, often, sometimes or never) and frequency of feeling pressure to lose weight by mother, father, siblings, partner/husband, female friends and male friends (almost always, often, sometimes or never). Responses for the last two questions were collapsed into the following two response options for data analysis purposes: almost always/often and sometimes/never.

Assessment of physical activity was based on items taken from the 16-item Baecke questionnaire of habitual physical activity (Beacke et al, [29]). These items included a question on whether a sport is played or exercise done (yes or no); if yes, the most and second most frequently performed sport or exercise done was asked. Questions on the number of hours spent per week on playing a particular sport or doing a particular exercise and the number of months per year a particular sport was played or an exercise was done followed. The total number of hours exercised per week was computed using this information and was denoted as vigorous physical activity. A further question asked about the number of minutes spent walking and/or cycling per day to and from university, shopping and work. The minutes per day were converted to minutes per week and this type of activity was denoted as moderate intensity physical activity. Students were also asked to rate their physical activity as more active, the same, or less active than that of their peers.

Meal pattern was assessed as frequency of consumption of specified meals or snacks per week (breakfast, mid-morning snack, lunch, late afternoon snack, dinner and after dinner snack). Food choices were assessed using a non-quantified food frequency questionnaire developed by Seme [30] and adapted for the purposes of this study. Foods were categorized into the following groups: fruit and vegetables (vitamin C rich, deciduous and tropical fruits; tomatoes; orange, yellow, green, cruciferous and mixed vegetables either fresh, tinned or frozen); high fibre foods (legumes; brown, whole wheat or rye bread or rolls; whole wheat breakfast cereals and oats porridge); refined carbohydrates (white bread or rolls, low fibre breakfast cereals, rice and pasta); low-fat protein rich foods (low-fat red meat, chicken without skin, fish, low-fat and fat-free dairy products); high fat foods (high fat red meat; processed and tinned meats; chicken with skin; full cream dairy products; cheese; margarines, butter and lard; plant oils, peanut butter/peanuts and other nuts or seeds; fried foods; pies, sausage rolls and samoosas; organ meats and take outs); energy-dense snacks/drinks (sugar, chocolate, sweets, cake, biscuits or doughnuts, fizzy drinks, crisps, jams, syrups or honey); healthy fats (soft tub margarine and olive oil); unhealthy fats (hard brick margarine, butter or lard); healthy food choices (fruits, vegetables, high fibre and low-fat protein rich foods combined) and unhealthy food choices (high fat foods, energy dense snacks and drinks and refined carbohydrates combined). Participants were required to indicate their frequency of intake of food items in the food list during the preceding week (response categories: not at all, 1–3 times/week, 4–6 times/week, 1 time/day or 2 times/day). The total frequency of intake per day from each food group was then computed.

### Data collection procedures and quality assurance

Students were recruited on the campuses of the University of Cape Town, the University of the Western Cape and Stellenbosch University. Recruitment strategies included presenting the study, described as a survey of weight-loss strategies of female students, to students in the residences (either orally or via posters and pamphlets) and also approaching students at university cafeterias and popular areas on campus. Students who volunteered to participate completed the consent forms and subsequently completed the questionnaire and height and weight measures.

Fieldworkers were trained and standardized to measure height and weight by a Level 3 antrhopometrist registered with the International Society for the Advancement of Kinanthropometry (ISAK). As the questionnaire was self-administered, the role of fieldworkers in this regard was to supervise. They checked each questionnaire for completeness in the presence of the student. Permission to recruit students on the university campuses was obtained from the relevant authorities at each university.

### Statistical analysis

All data analyses were conducted using *STATISTICA* 12, with the exception of the Kappa statistic and regression analyses that were conducted using STATA 11.0 (StataCorp LP, Texas). Weight loss practices used by dieters were described as frequencies (aim 1). Comparison of these practices between dieters with a BMI (kg/m^2^) <25 (normal weight) and those with a BMI (kg/m^2^) ≥25 (overweight/obese) was conducted using the Pearson’s Chi-square test. Comparison of characteristics between dieters and non-dieters (aim 2) was conducted using cross-tabulations and the Pearson’s Chi-square for race, mother’s level of education, accommodation, prior pregnancy, smoking, BMI category, satisfaction with weight and body shape, physical activity levels compared to that of pears and environmental influences. The independent samples *t*-test or Mann-Whitney *U*-test was used to compare age (years), weight (kg) height (m), BMI (kg/m^2^), frequency of intake of meals (times per week) and choices from food groups (times per day) and vigorous and moderate physical activity (minutes per week) between dieters and non-dieters depending on the distribution of these variables. The Kolmogarov-Smirnov and Lilliefors test was used to test for normality of these continuous variables. Agreement between actual BMI category (underweight, normal weight or overweight/obese) and perceived weight (too thin, just right or too chubby) was determined using the Kappa Statistic. To investigate predictors of BMI in the total group, the association between all study variables and a) BMI and b) being overweight/obese [BMI (kg/m2) ≥25] was determined using the Spearman correlation coefficient, Pearson’s Chi-square test, independent samples *t*-test or Mann-Whitney *U*-test as applicable. All variables that were significantly associated with BMI and/or being overweight/obese [BMI (kg/m2) ≥25] were included in a generalized linear regression model (coefficients reported) or logistic regression model [odds ratios (OR) reported], respectively. A *p*-value of <0.05 indicated statistical significance.

## Results

### Weight-loss strategies used by dieters

The study included 250 students of whom 162 (64.8 %) had attempted to lose weight in the previous year (dieters) and 88 (45.2 %) had not attempted weight loss in the past year (non-dieters). The socio-demographic profile and other characteristics of the sample are presented as part of the results on the comparison of weight related characteristics between dieters and non-dieters.

Healthy weight-loss strategies were commonly used by the dieters (*n* = 162). The most commonly used healthy practice was exercising more (89.5 %), followed by eating less sweets or drinking less sugar-sweetened beverages (82.1 %), eating more fruit and vegetables (82.1 %) and cutting out fats and fatty foods (75.3 %). The proportions of dieters who used unhealthy weight-loss strategies were 37.0 % for eating little food, 23.5 % for skipping meals 13.0 % for fasting, 9.3 % for using diet shakes and 4.3 % for smoking more cigarettes. Extreme weight-loss strategies were taking diet pills or herbal mixtures (21.6 %), vomiting (4.3 %), using laxatives (3.7 %) and the drug methamphetamine (0.6 %). There were no significant differences between BMI groups (BMI < 25 or BMI ≥25kg/m^2^) for use of any of these weight-loss methods.

The weight loss product used most commonly by dieters (*n* = 166) was Herbex (11.7 %). Other weight-loss products used by the dieters were Herbalife products (7.4 %), GI Lean products (5.1 %), Hoodia (4.3 %), USN weight-loss products (3.7 %), CLA products (3.1 %), Leanor (1.2 %) and Simply slim (0.6 %), Phentermine (0.6 %) and Sibutramine (1.2 %). Use of Herbex was significantly higher in the ≥25 kg/m^2^ BMI group than in the <25 kg/m^2^ group (22.9 % vs. 7 %) (Pearson’s Chi-square *p* = 0.009). There were no significant differences between the two BMI groups for use of any of the other products. Products not used by any student include Biomix Slimming Solution, Orlistat and products containing Amfepramone, Phendimetrazine and Cathine.

The most popular season for using weight-loss products (*n* = 110) was summer (50.9 %), followed by winter (23.6 %), spring (22.7 %) and autumn (3.7 %). There were no significant differences between the two BMI groups for this variable.

### Comparison of weight-related characteristics between dieters and non-dieters

#### Age and socio-demographic profile

The mean ages and socio-demographic profile of the dieters and non-dieters are described in Table 1. The race profile did not differ significantly between the two groups, with approximately half in both groups being white, a quarter black and the rest either mixed ancestry or Indian. Students in the two groups were equally likely to have a mother with tertiary level education, with the profile for accommodation either in a university residence, or in a private residence, or at home also being similar. Only two students reported a past pregnancy, one in each group and smoking was reported by 11.1 % dieters and 10.2 % non-dieters.

**Table 1:** Comparison of socio-demographic variables between dieters and non-dieters

	Dieters^a^		Non-dieters^b^		*P* values
Number	*n* = 162		*n* = 88		
Age mean(SD)	21.0 (1.7)		20.9 (1.8)		0.910^c^
Ethnicity (column %)					
Black		25.3		20.5	0.067^d^
White		52.5		53.4	
Mixed ancestry		15.4		25.0	
Indian		6.8		1.1	
Mother’s education (column %)					
Secondary school or less		40.1		53.3	0.064^d^
Tertiary qualification		59.9		47.7	

*SD* standard deviation

^a^Attempted weight loss in the past year

^b^Did not attempt weight loss in the past year

^c^Independant sample’s *t*-test

^d^Pearson’s Chi- square test

#### Anthropometric profile

The anthropometric profiles of the students are presented in Table 2. The mean BMI for both dieters and non-dieters was in the normal range. Compared with the non-dieters the dieters were significantly more likely to be overweight or obese and less likely to be underweight (Table 2). However, two thirds of the dieters were in the normal weight range. We conducted a power estimation for comparison of dieters (*n* = 162) with non-dieters (*n* = 88) using the mean ± SD BMI (kg/m^2^) for each group (Table 2) and the Power Mean Difference option of the OpenEpi calculator (Confidence Interval 95 %). A power of 99.9 % was indicated.

**Table 2:** Comparison of anthropometric variables between dieters and non-dieters

Anthropometrics	Dieters^a^		Non-dieters^b^		*P* values
Number	*n* = 162		*n* = 88		
Weight (kg) Mean (SD)	63.2 (10.7)		58.1 (9.3)		<0.001^c^
Height (m) Mean (SD)	1.6 (0.06)		1.64 (0.07)		0.467^c^
BMI kg/m^2^ Mean (SD)	23.9 (3.9)		21.7 (3.2)		<0.001^c^
BMI Category (column %)					
Underweight		1.2		10.2	<0.001^d^
Normal weight		69.1		78.4	
Overweight		24.1		11.4	
Obese		5.6		0	

*SD* standard deviation

^a^Attempted weight loss in the past year

^b^Did not attempt weight loss in the past year

^c^Indpendent samples *t*-test

^d^Pearson’s Chi-square test

#### Relationship between weight perception and actual BMI

It is evident that agreement between weight perception and classification according to actual BMI category was better among the non-dieters than the dieters (Table 3). Dieters were prone to see themselves as larger than they actually were, with the proportion of normal weight students who viewed themselves as “too chubby” being approximately three times higher among dieters than non-dieters. The proportion of overweight/obese students who perceived themselves to be normal weight, was similar in the two groups.

**Table 3:** Agreement between weight perception and classification according to BMI category for dieters and non-dieters (column %)

Weight perception	Dieters*			Non-dieters**		
	Under- weight	Normal	Overweight/obese	Under- weight	Normal	Overweight/obese
Number	*n* = 2	*n* = 112	*n* = 46	*n* = 9	*n* = 69	*n* = 10
“Too thin”	0^a^	1.8	0	22.2^a^	4.4	0
“Just right”	100	69.4^a^	18.8	77.8	85.5^a^	20.0
“Too chubby”	0	28.6	81.2^a^	0	10.1	80.0^a^

^a^Reflects correct perception of actual weight

*Attempted weight loss in the past year

**Did not attempt weight loss in the past year

Kappa statistic: Diet group*: value: 0.43; agreement: 51%; *p* < 0.001; Non-diet group**: value: 0.41; agreement 63.1%; *p* < 0.001

#### Satisfaction with weight and body shape

Compared with the non-dieters (*n* = 88) the dieters (*n* = 162) were significantly more likely (Pearson’s Chi-square *p* < 0.01) to be unsatisfied with their weight (53.1 % vs. 21.6 %), waist (43.2 % vs. 20.5 %), hips (45.1 % vs. 21.6 %), legs (47.5 % vs. 27.3 %) and arms (33.3 % vs. 10.2 %). There were no significant differences between the two groups for satisfaction with height (79.5 vs. 81.0), face (88.5 vs. 90.7) and buttocks (56.1 vs. 63.2).

#### Environmental influences

Results for dieters (*n* = 162) compared to non-dieters (*n* = 88) for the two questions in this section are as follows (please note that often/always for each group is reported, the balance in each group answered sometimes/never):

##### Use of different sources of information on weight management information

The Internet 45.1 % dieters vs. 26.1 % non-dieters (Pearson’s Chi-square *p* = 0.003); friends 40.1 % vs. 26.1 % respectively (*p* = 0.027); family 32.1 % vs. 18.1 % respectively (*p* = 0.018); and magazines 30.3 % vs 14.8 % respectively (*p* = 0.007). Use of the television, a nutrition expert, a physician, or a pharmacist did not differ between dieters and non-dieters. Use of television was 20.4 % for dieters vs. 18.2 % for non-dieters, a nutrition expert 11.7 % vs. 6.8 % respectively, a physician 11.7 vs. 5.7 % respectively and a pharmacist 5.7 vs. 2.3 % respectively.

##### Experience of pressure to lose weight from individuals in the environment

The mother 30.8 % dieters vs. 9.6 % non-dieters (Pearson’s Chi-square *p* < 0.001); father 21.2 % vs. 7.3 % respectively (*p* = 0.006); siblings 29.8 % vs. 15.3 % respectively (*p* = 0.012); husband/partner 21 % vs. 6.9 % respectively (*p* = 0.017); female friends 29.6 % vs. 9.1 % respectively (*p* < 0.001); and male friends 15.3 % vs. 4.7 % respectively (*p* = 0.013).

#### Physical activity

Dieters (*n* = 162) spent significantly more time participating in vigorous physical activity than non-dieters (*n* = 88) [55.4 (13.8, 115.4) vs. 18.5 (0, 55.4)] minutes per week; Mann-Whitney *U* test *p* < 0.001). Moderate intensity physical activity (e.g. walking) did not differ between the two groups, with the median (IQR) for both the dieters and non-dieters being 161 (63; 266) minutes per week. Furthermore, dieters were significantly more likely to think that they are more active than peers than non-dieters with the percentage more active, the same, or less active in the diet group being 33.5 %, 29.2 % and 37.3 % respectively vs. 18.4 %, 31 % and 50.6 % respectively in the non-diet group (Pearson’s Chi-square *p* = 0.03).

#### Meal patterns and food choices

Current weekly frequency of breakfast consumption was significantly higher in the dieters (*n* = 162) [7 (4; 7)] compared to the non-dieters (*n* = 88) [5 (4; 7)] (Mann-Whitney *U* test *p* = 0.042). There were no significant differences for other meals, with a midmorning snack being consumed 4 (2, 7) times per week by dieters and 4 (2, 5.5) by non-dieters, lunch 7 (7, 7) and 7 (7, 7) times per week respectively, an afternoon snack 5 (2, 7) and 4 (2, 6.5) times respectively, supper 7 (7, 7) and 7 (7, 7) times respectively and a snack after supper 2 (0, 5) and 2 (0, 4) times respectively.

Results for the comparison of current frequency of choices from investigated food groups between dieters and non-dieters are presented in Table 4. The dieters had a significantly higher frequency of intake of healthy food choices, specifically fruit and vegetables and low-fat protein-rich foods, while they had a significantly lower frequency of intake of unhealthy food choices, specifically refined carbohydrates and energy-dense snacks and drinks.

**Table 4:** Comparison of current daily frequency of food choice groups median (IQR) between dieters and non-dieters

Food choice group	Dieters^a^		Non-dieters^b^		*P* value^c^
	*n* = 162		*n* = 88		
Fruit & vegetables	2.6	(1.6, 3.7)	1.9	(1.2, 2.9)	0.007
High fibre foods^d^	1.0	(0.5,1.6)	1.0	(0.6, 1.6)	0.580
Low-fat protein-rich foods	1.4	(0.7, 2.1)	1.1	(0.6, 1.6)	0.017
Energy-dense snacks & drinks	2.0	(1.3, 3.1)	2.6	(1.9, 4.1)	<0.001
Refined carbohydrates	0.9	(0.3, 1.4)	1.3	(1.0, 1.7)	<0.001
High-fat foods	1.6	(0.9, 2.4)	1.9	(1.1, 2.9)	0.018
Healthy fats	0.6	(0.3, 1.0)	0.6	(0.3, 1.0)	0.976
Unhealthy fats	0	(0, 2.0)	0	(0, 2.0)	0.085
Healthy choices	5.6	(4.3, 7.9)	4.6	(3.6, 6.4)	0.006
Unhealthy choices	5.6	(3.3, 8.7)	7.6	(5.3, 10.2)	<0.001

*IQR* inter-quartile range

^a^Attempted weight loss in the past year

^b^Did not attempt weight loss in the past year

^c^Mann-Whitney *U*-test

^d^Exclluding fruit and vegetables

#### Predictors of BMI

All variables found to be associated with BMI on the one hand, and being overweight/obese (BMI ≥25kg/m^2^) on the other were considered in the two regression models constructed to identify possible predictors of BMI and weight category in the study sample (Table 5). These significantly associated variables included race; attempted weight loss in the year preceding the study (dieter vs. non-dieter); satisfaction with waist, hips, arms, legs, and stomach; physical activity compared to friends; pressure to lose weight from mother, father, siblings, and male friends; frequency per week of consumption of a mid-morning snack and daily frequency consumption of high-fat foods. Significant predictors for a higher BMI (continuous variable) and being overweight/obese (categorical variable) are presented in Table 5. These include: (a) for a higher BMI only: being a dieter; (b) for both a higher BMI and increased risk for BMI ≥25 kg/m^2^: black ancestry, dissatisfaction with one’s waist, currently perceiving oneself as being “chubby”, and a lower frequency of eating a mid-morning snack; and (c) for an increased risk for a BMI ≥25kg/m^2^ only: a higher frequency of consuming high-fat foods.

**Table 5:** Predictors of BMI in the total group of students (*n* = 250)

Outcome: BMI as a continuous variable^a^					Outcome: BMI ≥25^b^			
	Coefficient	*p*-value	95 % Confidence interval		Odds ratio	*p*-value	95 % Confidence interval	
Diet group								
No	0.00							
Yes	0.79	0.044	0.02	1.57	NS	NS	NS	NS
Race								
Black	0.00				1.00			
White	-2.31	<0.001	-3.19	-1.42	0.21	0.002	0.08	0.57
Other^c^	-1.85	<0.001	-2.88	-0.81	0.40	0.093	0.14	1.16
Satisfied with waist								
Yes	0.00				1.00			
Unsure	0.02	0.966	-1.04	1.09	2.16	0.262	0.56	8.33
No	1.53	<0.001	0.72	2.35	4.08	0.002	1.69	9.83
Current weight perception								
Not chubby	0.00				1.00			
Chubby	2.87	<0.001	2.05	3.70	13.22	<0.001	5.63	31.07
Mid-morning snack								
Days per week	-0.19	0.010	-0.34	-0.05	NS	NS	NS	NS
High-fat foods								
Frequency consumed per day	NS	NS	NS	NS	1.40	0.043	1.01	1.93
Constant	23.23	<0.001	22.18	24.28	0.04	<0.001	0.01	0.16

*NS* Not significant

^a^GLM regression; AIC = 4.93; BIC = 549.37

^b^Logistic regression; Log likelihood = -80.70

^c^Coloured and Indian combined

## Discussion

This research set out to investigate weight loss strategies used by female students and to compare weight management characteristics between dieters and non-dieters. Dieting during the past year was found to be prevalent among normal weight, overweight and obese students. Weight loss strategies used were mostly healthy, although unhealthy and extreme strategies were also used. Significant differences in weight, BMI, body weight perception and satisfaction, weight management related environmental influences, physical activity, meal pattern and food choices between dieters and non-dieters were evident.

Dieters and non-dieters were of equal age, with 24.1 % of dieters being overweight and 5.6 % obese, while 11.4 % of the non-dieters were overweight and none obese. For both groups these figures are below the national average reported for 15 to 24 year old females, namely 25.3 % being overweight and 21.7 % obese [3]. For dieters the proportion is higher than the 10 % white female students reported to be overweight and 0.8 % to be obese by Cilliers et al. [27], and in line with the 22.1 % black female students reported to be overweight/obese by Steyn et al. [31].

A history of dieting during the past year was present in almost two thirds of the total group of students. It is possible that the recruitment strategy, namely asking students whether they would be interested in participating in a study on the “dieting strategies of students”, created the perception that the study was focused on dieters only. However, dieting in the past year or two has been found to be a common phenomenon among female students at tertiary institutions in South Africa and in other countries. Data for South Africa reported that 55.3 % of white female students had attempted weight loss in the previous year [28], while the proportion was 42 % in black female students over the past two years [12]. Studies carried out in several other countries have also reported that a high proportion of young adult females, including many who have a normal weight, are trying to lose weight [25, 32, 33]. For example, Fayet et al. [34] reported a prevalence of 43 % for female Australian students, Lee [35] 41 % for non-overweight Korean female adolescents, and Schembre et al. [36] 76 % for white and 50 % for Native Hawaiian female students. This suggests that our sample was reasonably representative of female students, though there may have been some degree of over-sampling of dieters.

The most commonly (>75 % of dieters) used weight-loss methods were healthy, namely increasing exercise, increasing intake of fruit and vegetables, and reduced consumption of fat-rich foods, sweets and sugar-sweetened beverages. Unhealthy weight-loss methods, such as eating little food, skipping meals and fasting, were also commonly used (from 37 % to 13 % of dieters), which is in accordance with results from other studies, with eating less food and skipping meals generally being more commonly used than fasting [8, 12, 15, 25, 37]. Extreme weight loss strategies were also used by students in this study, but the prevalence was less than 5 % for each. The prevalence of using laxatives found in our study (4.1 %) is in line with previous research in female students, where it was reported to range from 1.6 % [8] to 15 % [12]. The prevalence of vomiting was found to be 3.4 % in our student sample, which is also similar to the prevalence of use of this strategy in other groups of students. For example, Malinauskas et al. [25] found that 4 % to 6 % of female college students used vomiting as a weight-loss method. Similar results were found by Neumark-Sztainer and Hannan [15] (6.8 %) and Alvarenga et al. [17] (3.3 %). Senekal et al. [12] reported that 11.7 % of their sample of South African black female students indicated that they used vomiting as a weight loss method.

Weight-loss products were most likely to be used in spring or summer, leaving room for speculation that dieting and use of these products in female students may be linked to getting in “form” for the summer period. The most commonly used weight-loss products were in descending order (≤11 % of dieters): Herbex, Herbalife, GI Lean products, Hoodia, USN products and CLA products. Senekal et al. [12] reported similar proportions of use of diet formulae/milkshakes and appetite suppressants by black South African female students. The use of Herbex was significantly higher in the overweight/obese sub-group. Use of commercial weight loss products may reflect increased pressure felt by overweight/obese individuals to lose weight. However, with very few exceptions these weight loss products suffer from a serious lack of supporting evidence [38, 39].

Comparison of weight related characteristics between dieters and non-dieters showed that the race profile was similar, with just more than two thirds of black participants being dieters. This may reflect acculturation in black female students, namely increased acceptance of the Western thin beauty ideal. These results confirm the notion of acculturation in black female students and women as discussed by Senekal et al. [12] and Puoane et al. [19, 24].

Dieters had a significantly higher BMI and prevalence of overweight and obesity than non-dieters. However, it is important to note that the weight of two thirds of the dieters was in the normal range. This suggests that dieters may have been successful with their weight loss attempt(s) or that they may desire to have a weight at the low end of normal or in the underweight range. Conversely, not all overweight young women seem motivated to lose weight as a number of the non-dieters in this study were overweight, which may be linked to the acceptance of a larger body weight in certain cultures [19–21]. Dieters in this study were more prone than non-dieters to see themselves as heavier than their actual weight, with normal weight students who viewed themselves as “too chubby” being approximately three times higher among dieters than non-dieters. Dieters were also less satisfied with their bodies as they were significantly more likely to be unsatisfied with their weight, waist, hips, legs and arms than non-dieters. Consistent with our findings, especially for the dieters, other studies have also observed that many young adult women have a distorted body-image [27, 33, 34, 40]. These results emphasize the possibility that normal-weight students may be engaging in unnecessary weight-reduction practices to ‘normalise’ their weight, increasing the risk for development of eating disorders. On the other hand, overweight students in both groups who perceive to have a normal weight, are at increased risk of development of obesity as they will not be motivated to lose weight.

Sources of weight management information and pressure to lose weight were investigated as weight management-related environmental characteristics of students. Dieters were significantly more likely to have used the Internet, friends, family and magazines (in descending order of use by dieters) than non-dieters for weight-management related information. Use of the television, a nutrition expert, a physician or pharmacist (in descending order of use by both groups), did not differ between dieters and non-dieters. This is in line with the findings of Steele and Senekal [41], namely that South African university students use family and friends, doctors and advertising as main sources of information on the need for dietary supplementation. Although dietitians and nutritionists are the experts in nutrition, students seem to lean towards informal and easily accessible sources of nutrition and weight management related information, which may not necessarily be evidence based. However, the potential advantages of using the Internet as information source should not be completely discounted. In their qualitative work in 11-to-19 year-old adolescents Gray et al. [42] identified such advantages, namely: avoiding a visit to a health professional, information being generally current as most websites are regularly updated (more so than is the case with print media), allowing an individual to search in ways unconstrained by place and time, being able to print and store information, personalization through feedback loops and facilitating the use of a social support network.

Pressure to lose weight from mothers, siblings and female friends, and to a lesser extent from the father and husbands/partners was experienced by both dieters and non-dieters. However, dieters were significantly more likely than non-dieters to have experienced pressure from all the mentioned individuals. These findings are similar to those of McCabe and Riciardelli [43] in 14-year-old girls, namely that the strongest influences on weight-loss decisions were mothers and best female friends, with much less influence coming from fathers or the media. This is especially important considering the findings by Peterson et al. ([13], p. 636) that: “Maternal pressure to lose weight and to be attractive appeared to have a strong influence on adolescents’ view of their physical appearance. These nonbehavioral, psychological influences may be firmly established from mother input, perhaps from an early age, to such an extent that even the young person’s friends are unable to affect them.”

Dieters in our sample spent almost three times more time doing vigorous physical activity (sport participation or exercise) while the time spent doing moderate physical activity (e.g. walking) was the same. Dieters were also significantly more likely to perceive themselves to be more active than their peers. The current guidelines for physical activity in the United States of America are that adults should spend at least 150 min per week engaged in moderate-intensity or 75 min per week of vigorous-intensity aerobic physical activity [44]. When time spent in vigorous and moderate physical activity is summed in each of the two groups, it is evident that both groups met the physical activity requirements for health. The higher level of participation in vigorous physical activity by dieters may be the result of increased awareness of the importance of physical activity in weight control, as increased physical activity was one of the dieting strategies most commonly mentioned by these students.

Dieters made unhealthy food choices that included energy dense snacks and drinks, refined carbohydrates and high fat foods significantly less often, and healthy food choices that included fruit/vegetables, wheat fibre and legumes and low-fat protein-rich foods significantly more often than non-dieters. Dieters may have been more aware of healthy food choices, as is reflected in the fact that increased intake of fruit/vegetables and decreased intake of energy-dense foods were very commonly mentioned as weight-loss strategies. However, it needs to be noted that neither dieters nor non-dieters met the South African Food-Based Dietary Guideline (SAFBDG) [45] for eating five or more fruits and vegetables (combined) per day. This is in line with the finding by Mckize et al. [46] who conducted a review of dietary surveys in the adult South African population, that two of the three most commonly deficient food groups are fruit and vegetables, with dairy being the third (dairy was not assessed as an distinct group in this study). Further support for this dietary risk in adult South Africans comes from the SANHANES [3], where it was found that only 4.5 % of the sample consumed four or more fruits and vegetables (combined) per day. Results show that students in our study may also not be meeting the guideline “eat sugar and fat sparingly,” as unhealthy (energy-dense) food choices were made either equally frequent (dieters) or more frequently (non-dieters) than healthy food choices. The fact that a higher frequency of high-fat food choices increased the risk for overweight/obesity in our sample (risk increased by 40 % for each additional time per day a high-fat food item was consumed) confirms the obesity risk denoted by the consumption of energy-dense foods.

The meal pattern of dieters and non-dieters in our study is in line with the SAFBDG recommendation that food intake should be spread throughout the day in the form of regular small meals [45]. Dieters consumed breakfast significantly more frequently than non-dieters, possibly also reflecting better awareness of healthy eating guidelines. It is interesting to note that a lower frequency of intake of a mid-morning snack predicted a higher BMI, supporting the notion that a pattern of more frequent, smaller meals supports achieving and/or maintaining a healthy weight.

Black students in our study were found to be at increased risk for a higher BMI and being overweight/obese when compared to white and coloured/Indian students. This is in line with data from SANHANES, namely that the prevalence of overweight/obesity is highest in black women of varying ages [3]. Further predictors for a high BMI and increased risk for overweight/obesity found in our study include not being satisfied with one’s waist and currently perceiving one as being too “chubby”, which could be correct or the result of a distorted body image. Attempted weight loss during the past year predicted BMI, but not risk for overweight/obesity, implying that dieting increases with increasing BMI, but that those who diet are not necessarily overweight/obese. This finding supports the notion that the decision to diet may not necessarily be prompted by being overweight/obese and the need for improved health, but rather by the wish to be thin consistent with the common Western ideal of beauty.

The findings of this research need to be viewed in the context of the study limitations. These include the cross-sectional design, the convenience sample drawn from select South African universities, which is not representative of the female student population in South Africa, and the use of a self-administered questionnaire, which leaves room for error in the responses given. Furthermore, more comprehensive physical activity and dietary intake assessment may have provided more in depth insights in the differences between dieters and non-dieters for these variables.

Bearing these limitations in mind it can be concluded that the findings reported here show that weight-loss attempts may be highly prevalent in female students regardless of weight status and race. Although healthy weight loss strategies seem to be commonly used, unhealthy and extreme methods, including a variety of commercial weight loss products, are also used. When compared to non-dieters, dieters are characterized by a higher BMI (although not necessarily in the overweight/obese range); a higher prevalence of overweight and obesity (although the majority are normal weight); body image distortions (normal weight students perceiving themselves to be overweight, although some overweight students perceive themselves to be normal weight); dissatisfaction with weight, arms, legs, hips and stomach; pressure experienced from especially mothers, friends and siblings to lose weight; use of the Internet, friends, family and magazines for weight-management information and a healthier life-style i.e. higher levels of physical activity that meet the physical activity guidelines and a higher frequency of healthy and lower frequency of unhealthy food choices, although not yet meeting the FBDG. Finally, weight loss attempts during the past year predict BMI, but not risk for overweight/obesity, implying that dieting prevalence increases with increasing BMI, but those who diet are not necessarily overweight/obese.

Healthy weight management, whether it involves weight gain for those who are underweight, prevention of weight gain for those who are normal weight, weight loss or prevention of further weight gain for those who are overweight/obese or weight maintenance for those who have lost weight, is core to ensuring optimal health and prevention of eating disorders, non-communicable diseases [11]. Female students are the mothers and caregivers of tomorrow and their weight management practices from pregnancy onwards hold strong potential to set the stage for the weight and ultimately the health of future generations.

## Conclusions

The results of this research leave no doubt that the following should be emphasized in healthy weight management interventions aimed at female students: education regarding body shape perceptions, reasonable weight goals, when weight loss is essential, which weight loss strategies to use, hazards of unhealthy and extreme weight-loss methods, management of pressure by others to lose weight in those who are actually normal weight and critical consumption of weight management-related information from informal sources. At the same time there seems to be a need for nutrition experts to be more visible and active in the provision of relevant education and support for skills development to ensure optimal weight management practices in the general population.

## Abbreviations

BMI: Body mass index
GI: Glycemic index
USN: Ultimate sports nutrition
CLA: Conjugated linoleic acid
ISAK: International society for the advancement of kinanthropometry
SAFBDG: South african food-based dietary guidelines
SANHANES: South african national health and nutrition examination survey
